# A Case of Placenta Increta Mimicking Submucous Leiomyoma

**DOI:** 10.1155/2014/429406

**Published:** 2014-12-07

**Authors:** Ali Ekiz, Ibrahim Polat, Sezcan Mumusoglu, Burchan Aydiner, Cagdas Ozdemir, Hilal Serap Arslan

**Affiliations:** ^1^Department of Materno-Fetal Medicine, Kanuni Sultan Suleyman Education and Research Hospital, Turgut Ozal Street No. 1, Kucukcekmece, 34303 Istanbul, Turkey; ^2^Department of Gynecological Oncology, Istanbul Zeynep Kamil Maternity and Children Training and Research Hospital, Dr. Burhanettin Ustunel Street No. 10, Uskudar, 34668 Istanbul, Turkey; ^3^Department of Obstetrics and Gynecology, Kanuni Sultan Suleyman Education and Research Hospital, Turgut Ozal Street No. 1, Kucukcekmece, 34303 Istanbul, Turkey; ^4^Department of Pathology, Kanuni Sultan Suleyman Education and Research Hospital, Turgut Ozal Street No. 1, Kucukcekmece, 34303 Istanbul, Turkey

## Abstract

In recent years with the increase in cesarean section rates, the frequency of placenta accreta cases rises. It causes 33–50% of all emergency peripartum hysterectomies. We present a 42-year-old case who was caught with early postpartum hemorrhage due to retained placental products. The ultrasonography showed a 65 × 84 mm mass in the uterine cavity after the delivery. Due to presence of early postpartum hemorrhage which needs transfusion, an intervention decision was made. The patient underwent curettage but the mass could not be removed so that placental retention was ruled out. Submucous leiomyoma was made as first-prediagnosis. Hysterectomy operation was performed as a curative treatment. Placenta increta diagnosis was made as a final diagnosis with pathological examination. As a result, placental attachment disorders may be overlooked if it is not a placenta previa case.

## 1. Introduction

Placenta accreta is defined when the whole placenta or a part of it invades myometrium in an abnormal way. As the pathogenesis, defective decidualization during implantation is responsible. When the chorionic villi invade only the myometrium, the term placenta increta is used, whereas placenta percreta describes the invasion through the myometrium and serosa and rarely adjacent organs. In clinical practice, the general term “placenta accreta” is used to describe all 3 grades of abnormal placental attachment. The frequency of this life-threatening obstetric complication is responsible for out of 533 to 2510 childbirths [1, 2]. In recent years with the increase in cesarean section rates, the frequency of placenta accreta cases rises. It causes 33–50% of all emergency peripartum hysterectomies. Placenta previa is the most important risk factor for placenta accreta. The asset of placenta previa and previous cesarean exponentially increases the risk. A usually asymptomatic patient is suspected during obstetric ultrasonography with some findings. But here, atypically, a placenta increta case is presented with encountering early postpartum atony.

## 2. Case Presentation

42-year-old female patient's, Gravida 7, Parity 5, Abortion 1, previous births were spontaneous vaginal delivery and obstetric history was unremarkable. At the 40th week of pregnancy she underwent limited ultrasonographic evaluation: fundus located placenta and estimated fetus with a weight of 4000 g were identified; the additional examination was unremarkable. The patient is presenting with complaints of cramps, and as the result of the evaluation, she was admitted to the delivery room to be followed-up. Hematocrit was 31% during the hospitalization. The patient was living in the eastern region of Turkey, a rural region, that is why the antenatal follow-ups were inadequate. First examination of the patient was performed during the first trimester, as the detection of the pregnancy, and the second/last examination was held when the patient was admitted to the delivery room. In addition, we have not reached any record or report of ultrasonographic evaluation of pregnancy.

At 12th hour of admission, the patient gave birth to a 3900 g weight and 52 cm tall girl with normal spontaneous vaginal delivery. The placental delivery was uneventful and placental evaluation after delivery revealed normal appearance. There was no suspicion of lacking in cotyledons on maternal surface of the placenta. There was no sign for placental abruption. The patient was put in the service for follow-ups to be carried out without bleeding. Postpartum 4th hour of hemorrhage was treated with using uterotonic agents (oxytocin and methyl ergonovine) and the bleeding was stopped. The ultrasonography showed a 65 × 84 mm mass with irregular borders in the uterine cavity. Primarily submucosal fibroids or stalked leiomyoma came to mind, and placental retention was considered as the second preliminary diagnosis due to succenturiate placenta, although removed placenta has normal appearance. During the follow-ups intermittent heavy vaginal bleeding occurred and the family was informed for curettage to be taken on. However, we were unsuccessful during the curettage; the mass was rigid and was fixed in contrast with placenta so that placental retention was ruled out.

On the 2nd day of postpartum intermittent heavy vaginal bleeding continued. Despite of 4 units of packed red blood cells transfusion the hematocrit level was found to be 26-27%. Because of being unable to perform further radiological investigations and ongoing vaginal bleeding, surgery was offered to patient as a curative treatment. Families were informed and their consent was taken for surgery. The family, who does not request for fertility preservation, asked for hysterectomy.

**Figure 1:**
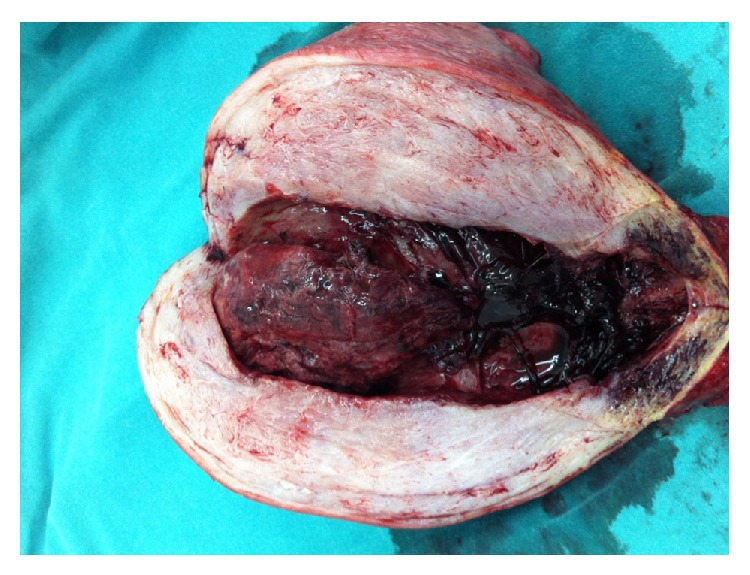
Macroscopy of uterus (hysterectomy material).

**Figure 2:**
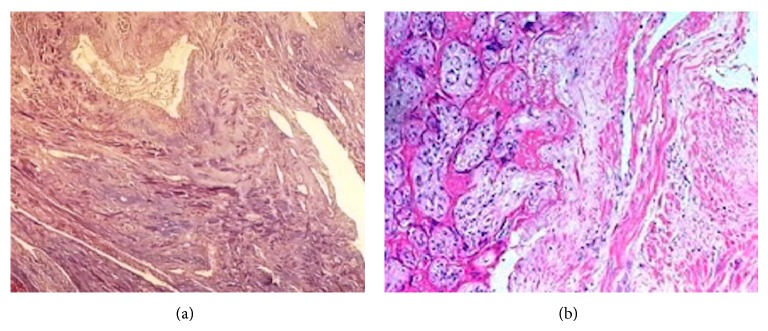
Chorionic villi invading the myometrium were shown with (a) Masson-trichrome staining and (b) hematoxylin-eosin methods.

In the operation a 20 week-size uterus was observed. When the uterine cavity is opened postoperatively, approximately 8 cm of mass filling the cavity was witnessed (Figure 1). On the 4th day of postpartum the patient, who was undergoing a smooth postoperative recovery period, was discharged with 28% hematocrit. In the presence of chorionic villi invading the myometrium on the pathology report, placenta increta was diagnosed (Figure 2).

## 3. Discussion

In the past 50 years with increasing cesarean rates, placental invasion abnormalities were observed 10 times more than usual [3]. Moreover, hysterectomy rate caused by placenta accreta increased by 23% between 1994 and 2007 [4]. Placenta previa and placenta accreta risk is 3% through the patients with previous caesarean section, while the risk increases to 40–67% with 3 or more cesarean sections previously [5]. Without previous uterine surgery, placenta previa and placenta accreta risk is 1–5% [6].

Clinically, patients with risk factors (especially both previous caesarean sections and placenta previa) are usually suspected for diagnosis antenatally. High maternal age, multiparity, myometrial damage (myomectomy, curettage, Asherman's syndrome, and endometrial ablation), and uterine artery embolization constitute as the other risk factors for placenta accreta. Patients without antenatal diagnosis may apply to emergency room with heavy vaginal bleeding or the diagnosis may be done by massive bleeding during placental separation after caesarean section or vaginal delivery. Bleedings may be life threatening.

The diagnosis usually starts with suspicion on antenatal follow-ups. In a patient with placenta previa the diagnosis is suspected with sonographic evaluation. Sonolucent areas in the placenta, loss of hypoechoic areas between placenta and myometrium, hypervascularization on the surface between serosa and bladder on Doppler ultrasound, and vascular stasis with turbulent flow are suggestive findings for placenta adhesion abnormalities sonographically. In the literature of the ultrasound are given sensitivity of 89.5% and specificity of 91% for the diagnosis of placenta accreta [7]. Magnetic resonance imaging (MRI) may be useful where sonography is insufficient. When placental invasion abnormalities are suspected, the family should be informed, and the birth should be in a tertiary center with preoperative preparations being carried out in a planned manner.

In the case presented here, cesarean delivery and placenta previa as the most important risk factors are not available. Although grand multiparity and high maternal age are counted as risk factors, it is not suspected of diagnosis of accreta antenatally. At this point, inadequate and improper antenatal surveillance may be the cause of missing the diagnosis. In the literature it is reported that, through the cases with five or more pregnancies, risk of accreta increases 3,9 times [8].

Uterine atony is the most common cause of early postpartum hemorrhage. Other common early postpartum hemorrhage reasons are upper and lower genital tract lacerations, lower urinary tract lacerations, and retaining placenta. Retaining placental products due to the placental adhesive disorders are rarely detected in cases with early postpartum hemorrhage. As it is presented, we can face patients with placental attachment disorder who are not diagnosed with placenta previa. In such rare cases, it is difficult to doubt and diagnose antenatally. The management of the cases encountered during operation or postpartum period is also rough and this situation increases the maternal morbidity and mortality. As in the case presented here, sometimes the discrimination cannot be made clearly in ultrasonographic examination.

In patients with postpartum hemorrhage the diagnosis of placental retention and placenta accreta should be kept in mind if the mass cannot be removed with curettage. In conclusion, the antenatal diagnosis seems to be the key point for management of placenta accreta.
